# The ESCRT-III protein VPS4, but not CHMP4B or CHMP2B, is pathologically increased in familial and sporadic ALS neuronal nuclei

**DOI:** 10.1186/s40478-021-01228-0

**Published:** 2021-07-19

**Authors:** Alyssa N. Coyne, Jeffrey D. Rothstein

**Affiliations:** 1 Brain Science Institute, Johns Hopkins University School of Medicine, Baltimore, MD 21205 USA; 2 Department of Neurology, Johns Hopkins University School of Medicine, Baltimore, MD 21205 USA

**Keywords:** Nucleoporins, POM121, Nuclear pore complex, ALS, FTD, CHMP7, ESCRT-III, VPS4, CHMP2B, CHMP4B

## Abstract

Nuclear pore complex injury has recently emerged as an early and significant contributor to familial and sporadic ALS disease pathogenesis. However, the molecular events leading to this pathological phenomenon characterized by the reduction of specific nucleoporins from neuronal nuclear pore complexes remain largely unknown. This is due in part to a lack of knowledge regarding the biological pathways and proteins underlying nuclear pore complex homeostasis specifically in human neurons. We have recently uncovered that aberrant nuclear accumulation of the ESCRT-III protein CHMP7 initiates nuclear pore complex in familial and sporadic ALS neurons. In yeast and non-neuronal mammalian cells, nuclear relocalization of CHMP7 has been shown to recruit the ESCRT-III proteins CHMP4B, CHMP2B, and VPS4 to facilitate nuclear pore complex and nuclear envelope repair and homeostasis. Here, using super resolution structured illumination microscopy, we find that neither CHMP4B nor CHMP2B are increased in ALS neuronal nuclei. In contrast, VPS4 expression is significantly increased in ALS neuronal nuclei prior to the emergence of nuclear pore injury in a CHMP7 dependent manner. However, unlike our prior CHMP7 knockdown studies, impaired VPS4 function does not mitigate alterations to the NPC and the integral transmembrane nucleoporin POM121. Collectively our data suggest that while alterations in VPS4 subcellular localization appear to be coincident with nuclear pore complex injury, therapeutic efforts to mitigate this pathogenic cascade should be targeted towards upstream events such as the nuclear accumulation of CHMP7 as we have previously described.

**Supplementary Information:**

The online version contains supplementary material available at 10.1186/s40478-021-01228-0.

## Introduction

Amyotrophic lateral sclerosis (ALS) is a devastating neurodegenerative disorder affecting upper and lower motor neurons within the brain and spinal cord. To date, mutations in more than 20 genetic loci have been identified as causative of familial ALS (fALS). Of these, a hexanucleotide repeat expansion (HRE) in the first intron of the *C9orf72* gene is the most common. However, the vast majority of ALS cases have no known genetic cause and are termed sporadic ALS (sALS) [3, 8, 9, 22]. Despite the heterogenous etiology, clinical and cellular pathologies and pathway disruptions of fALS and sALS are similar [13, 16, 18, 22]. While the genetic underpinnings and pathological hallmarks of ALS are increasingly well defined, the molecular mechanisms that lead to neuronal dysfunction and end-stage pathology remain understudied.

We have recently established that nuclear pore injury, as defined by the reduction of specific nucleoporins (Nups) from the nuclear pore complex (NPC) and nucleoplasm, is an early and significant pathologic event in sALS and *C9orf72* ALS/FTD [4, 5]. Importantly, loss of a subset Nups from the human neuronal NPC impacts functional nucleocytoplasmic transport (NCT), downstream TDP-43 function and cytoplasmic mislocalization, and ultimately neuronal survival in response to glutamate stress in human ALS models [4, 5]. Mechanistically, aberrant nuclear accumulation of the ESCRT-III pathway protein CHMP7 is sufficient to initiate this NPC injury which in turn leads to downstream neuronal dysfunction and pathology in sALS and *C9orf72* ALS/FTD [4, 5].

The ESCRT-III pathway functions to remodel membranes during multiple cellular processes including nuclear and plasma membrane repair, neuronal pruning, endosomal and exosomal trafficking, cell division, and multivesicular body formation. In total, there are multiple proteins including CHMP1-7 and VPS4, that have been identified as components of the ESCRT-III pathway [17, 29]. In yeast and non-neuronal mammalian cells, CHMP7 functions as an adapter for ESCRT-III mediated NPC and nuclear envelope (NE) surveillance and homeostasis [15, 23, 29, 32, 34]. In some non-neuronal model systems, nuclear localization of CHMP7 has been reported to subsequently recruit the ESCRT-III proteins CHMP4B and CHMP2B to sites of nuclear injury and Nup turnover [6, 25, 29, 30]. Ultimately, activation of this surveillance pathway culminates in scission and removal of NPCs, Nups, and NE components by the AAA-ATPase VPS4 [23, 29, 32–34]. We have previously shown that in human neurons, the proteins and pathways regulating the nuclear localization of CHMP7 are, in part, distinct from those identified in yeast [4, 23, 32, 34]. However, the contribution of other ESCRT-III proteins and the downstream effector VPS4 to CHMP7 mediated NPC injury in human ALS neurons remains unknown.

Here, using induced human pluripotent stem cell (iPSC) derived spinal neurons (iPSNs) and postmortem human tissues, we show that the nuclear expression of VPS4, but not CHMP4B or CHMP2B, is pathologically increased in *C9orf72* ALS/FTD and sALS human neurons. Further, the increase in nuclear VPS4 is dependent upon CHMP7 in ALS neurons. Consistent with a role as a downstream effector in ESCRT-III mediated NPC homeostasis, impaired VPS4 function is not sufficient to restore proper Nup distribution within the nucleus and nucleoplasm.

## Materials and methods

### iPSC derived neuronal differentiation

Mutant *C9orf72*, sALS, and non-neurological control iPSC lines were obtained from the Answer ALS repository at Cedars-Sinai (see Additional file 2: Table 1 for demographics) and maintained on Matrigel with MTeSR according to Cedars Sinai SOP. iPSCs were differentiated into mixed spinal neuronal populations using the direct induced motor neuron (diMNs) protocol as previously described [4, 5]. All cells were maintained at 37 °C with 5% CO_2_. iPSCs and iPSNs routinely tested negative for mycoplasma.

### ASO treatment of iPSNs

As previously described [4], on day 25 of differentiation, 5 μM scrambled control (676630): CCTATAGGACTATCCAGGAA or CHMP7 targeting (1508917): TGTTACCCTCAGATACCGCC ASOs were added to the culture media. Media and ASO were exchanged every 3 days until iPSN analyses were carried out on day 40 of differentiation. ASOs were generously provided by Ionis Pharmaceuticals.

### Nucleofection of iPSNs

On day 18 of differentiation, iPSNs were dissociated with Accutase following manufacturer protocol to assist with single cell dissociated and subjected to suspension based nucleofection using the Lonza P3 Primary Cell 4D Nucleofector Kit (Lonza) and program DC104. 5 million iPSNs and 4 μg plasmid DNA were used for each nucleofection reaction. Plasmids used are as follows: GFP (Addgene 54759), VPS4 GFP (Addgene 116924), and VPS4^E228Q^ GFP (Addgene 80351). Nucleofected iPSNs were plated in Matrigel (Corning) coated cell culture dishes and media was exchanged the next day and subsequently every 3 days until downstream analyses on day 40 of differentiation.

### Nuclei isolation and super resolution structured illumination microscopy

Nuclei were isolated from iPSNs and postmortem human motor and occipital cortex tissue using the Nuclei Pure Prep Nuclei Isolation Kit (Sigma Aldrich) following manufacturer protocol with slight modifications as previously described [4, 5]. About 10 million iPSNs or 100 mg of frozen postmortem motor cortex tissue (obtained from the Target ALS Human Postmortem Tissue Core (see Additional file 2: Table 2 for demographic information) was used for nuclei isolation. A 1.85 M sucrose gradient was used to enrich for neuronal nuclei. Following isolation, nuclei were centrifuged onto collagen coated (1 mg/mL; Advanced Biomatrix) slides with a CytoSpin 4 centrifuge (Thermo Fisher Scientific) and immunostained as previously described [4, 5] (see Additional file 2: Table 3 for antibody information). Isolated nuclei were subsequently imaged by super resolution structured illumination microscopy (SIM) using a Zeiss ELYRA S1 as previously described [4, 5]. All images were acquired using identical imaging parameters (e.g. laser power, gain) and subjected to default SIM deconvolution and processing in Zeiss Zen Black 2.3 SP1. Representative images are presented as 3D maximum intensity projections generated in Zeiss Zen Black 2.3 SP1. Images were faux colored green for contrast and display.

### Western blots

Nuclei and iPSN lysates were generated as previously described [4, 5] using RIPA lysis buffer. 5 μg protein was subjected to SDS-PAGE using 4–20% acrylamide gels (BioRad) and transferred onto nitrocellulose membranes with the Trans-Blot Turbo Transfer System as previously described [4, 5]. Following 30 min room temperature incubation in block (5% nonfat milk in 1X TBS containing 0.1% Tween-20), blots were incubated with rotation in primary antibody diluted in block overnight at 4 °C. See Additional file 2: Table 3 for antibody information. After ~ 16–18 h, blots were washed 4× 10 min with 1× TBST and then incubated with rotation in secondary antibody diluted in block for 1 h at room temperature. See Additional file 2: Table 3 for antibody information. Blots wee then washed 4× 10 min with 1× TBST and incubated with ECL substrate (Thermo Fisher Scientific, Millipore) for 30 s. The GE Healthcare ImageQuant LAS 400 system was used to acquire chemiluminescent images. Blots were incubated for 15 min at room temperature with 30% H_2_O_2_ to facilitate sequential probing without stripping [20]. Analysis was carried out with FIJI software. GAPDH and Lamin B1 were used for normalization.

### Statistical analysis

All data analysis was conducted with FIJI or Imaris as previously described [4, 5]. The analyzer was completely blinded to genotype/treatment/time point/brain region information. All statistical analyses were performed using Prism version 9 (GraphPad). For imaging experiments, statistical analyses were performed whereby the average of all nuclei or cells evaluated per each iPSC line, patient, and treatment condition represents n = 1. The total number of nuclei or cells evaluated per experiment is indicated in the figure legends. Two-tailed Student’s t-test, One-way ANOVA with Tukey’s multiple comparison test, or Two-way ANOVA with Tukey’s multiple comparison test was used as appropriate for experimental design and as indicated in figure legends. *p < 0.05, **p < 0.01, ***p < 0.001, ****p < 0.0001. Violin plots are used to display the full spread and variability of large data sets (> 10 data points). Center dotted line indicates median value. Two additional dotted lines indicate the 25th and 75th percentiles. Bar graphs with individual data points are used to display summary data sets with < 10 data points.

## Results

### Expression of the ESCRT-III protein VPS4 is increased in C9orf72 and sALS neuronal nuclei

**Fig. 1:**
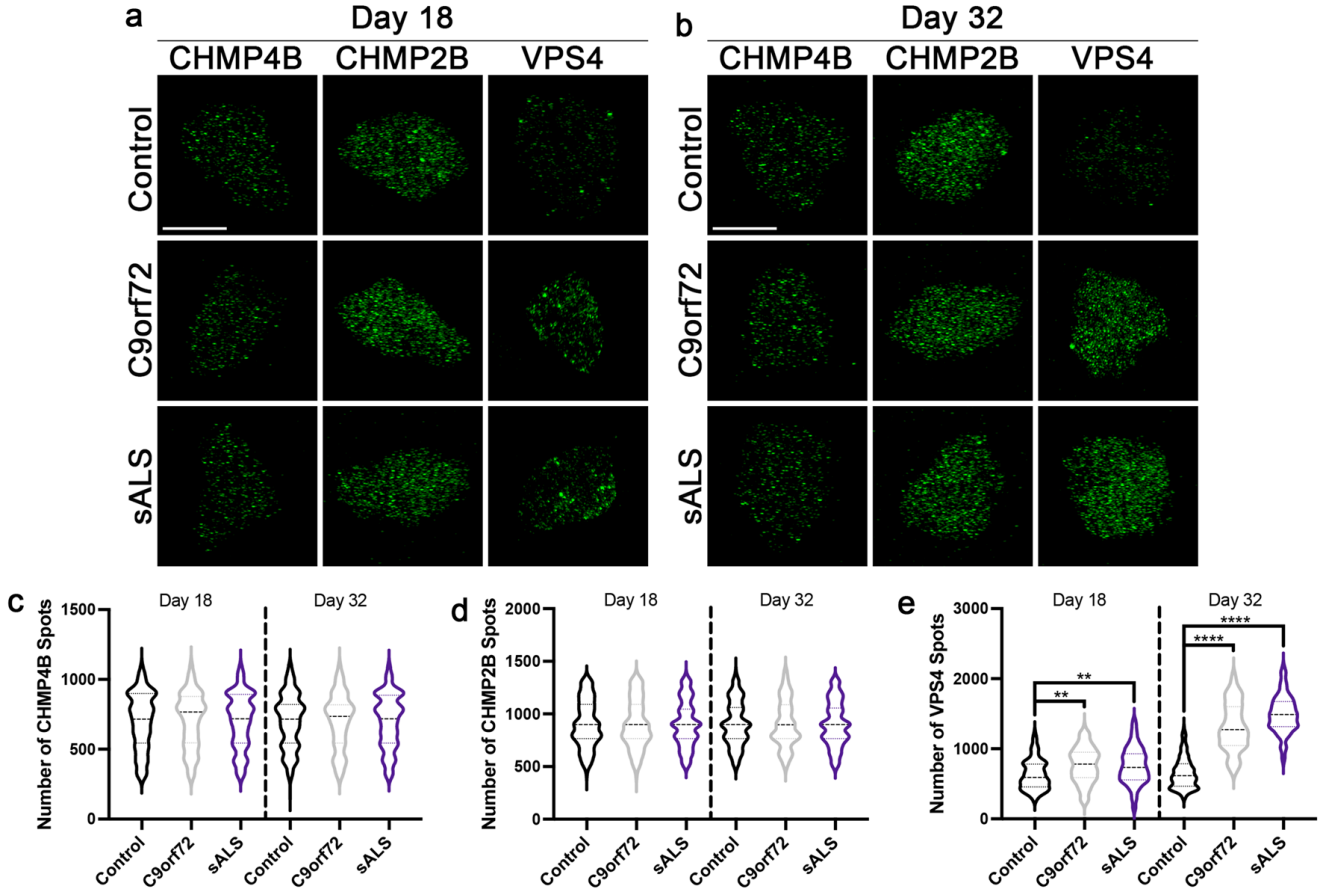
VPS4, but not CHMP4B nor CHMP2B, is increased in C9orf72 and sALS iPSN nuclei. **a**, **b** Maximum intensity projections from SIM imaging of CHMP4B, CHMP2B, and VPS4 in nuclei isolated from control, *C9orf72*, and sALS iPSNs at day 18 (**a**) and 32 (**b**) of differentiation. Genotype as indicated on left, time point and antibody as indicated on top. **c**–**e** Quantification of CHMP4B (**c**), CHMP2B (**d**), and VPS4 (**e**) spots. n = 10 control, 10 *C9orf72*, and 10 sALS iPSC lines, 50 NeuN + nuclei per line/time point. One-way ANOVA with Tukey’s multiple comparison test was used to calculate statistical significance. **p < 0.01, ****p < 0.0001. Scale bar = 5 μm

We have previously identified increased nuclear localization and expression of the ESCRT-III protein CHMP7 as an early and consequential pathologic event leading to NPC injury in *C9orf72* ALS/FTD and sALS [4]. Given the previously reported involvement of additional ESCRT-III proteins (CHMP4B, CHMP2B, and VPS4) in NPC and NE surveillance and homeostasis [6, 25, 29, 30], we sought to determine whether the nuclear distribution and cellular expression of CHMP4B, CHMP2B, and VPS4 was altered in ALS neuronal nuclei. Using immunostaining SIM, we found that the amount of VPS4, but not CHMP4B nor CHMP2B, was increased in nuclei isolated from *C9orf72* ALS/FTD and sALS iPSNs compared to controls (Fig. 1). Notably, similar to our published observations for CHMP7 [4], the increase in nuclear VPS4 spots occurred at a time point prior to the initiation of NPC injury (Fig. 1a, e). Importantly, we do not detect an overall increase in VPS4, CHMP4B, or CHMP2B levels in *C9orf72* ALS/FTD or sALS whole cell iPSN lysates (Additional file 1: Figure S1a–d). Highlighting the utility of SIM for evaluating not only protein distribution, but also expression, western blot analyses quantitatively confirmed an increase in VPS4, but not CHMP4B nor CHMP2B protein in nuclei isolated from *C9orf72* ALS/FTD and sALS iPSNs (Additional file 1: Figure S1e–h). Together, these data suggest that like CHMP7 [4], VPS4 may be relocalized from the cytoplasm to the nucleus in ALS neurons.

**Fig. 2:**
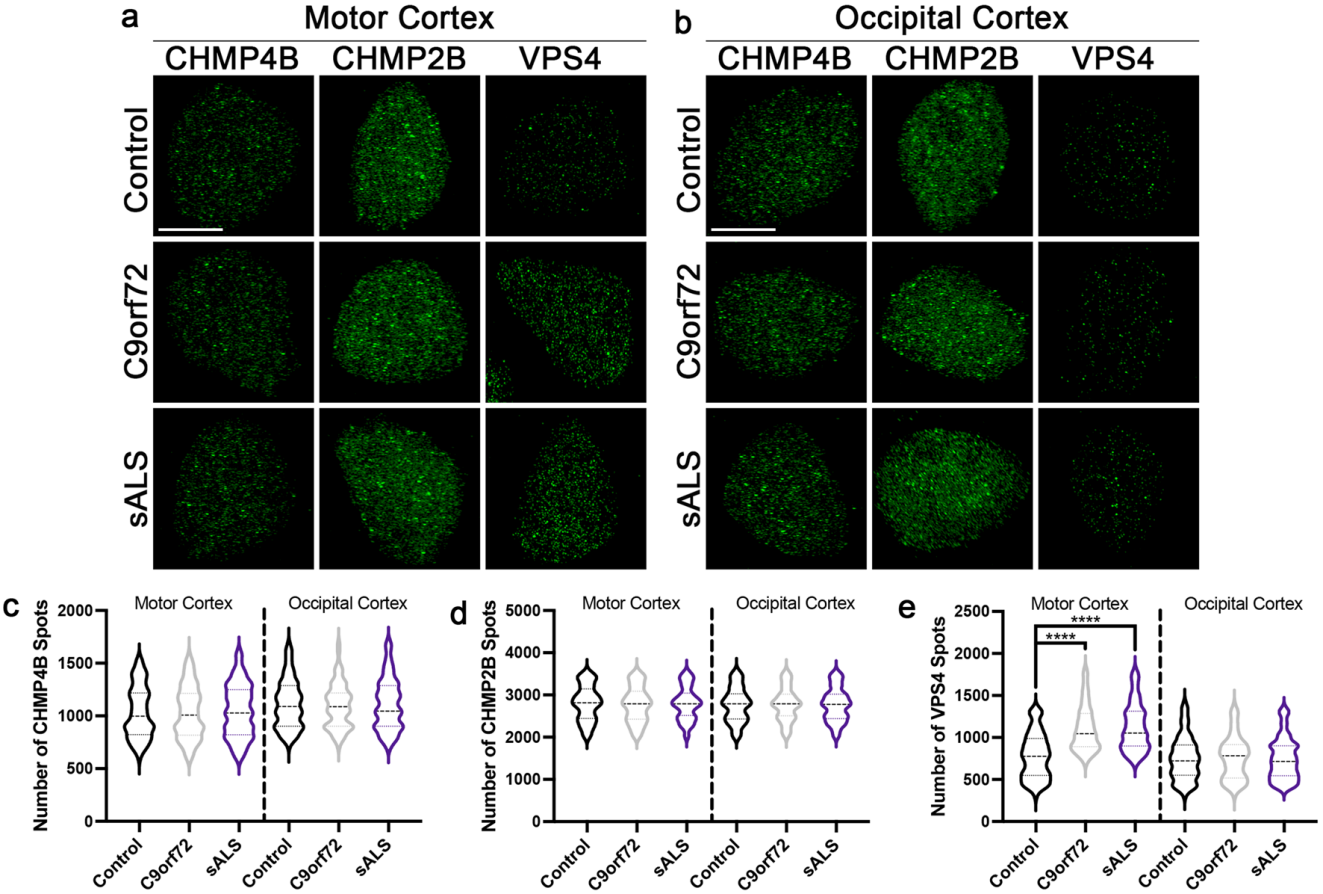
VPS4, but not CHMP4B nor CHMP2B, is increased in C9orf72 and sALS postmortem motor cortex neuronal nuclei. **a**, **b** Maximum intensity projections from SIM imaging of CHMP4B, CHMP2B, and VPS4 in nuclei isolated from postmortem control, *C9orf72*, and sALS motor (**a**) and occipital (**b**) cortex tissue. Genotype as indicated on left, brain region and antibody as indicated on top. **c**–**e** Quantification of CHMP4B (**c**), CHMP2B (**d**), and VPS4 (**e**) spots. n = 6 control, 6 *C9orf72*, and 9 sALS cases, 50 NeuN + nuclei per line/brain region. One-way ANOVA with Tukey’s multiple comparison test was used to calculate statistical significance. ****p < 0.0001. Scale bar = 5 μm

To validate that our results from iPSNs recapitulates observations in real human disease tissues, we performed immunostaining and SIM for CHMP4B, CHMP2B, and VPS4 in nuclei isolated from postmortem human motor and occipital cortex tissues. Consistent with our data in iPSNs, VPS4, but not CHMP4B nor CHMP2B, was increased in neuronal nuclei from *C9orf72* ALS/FTD and sALS motor cortex (Fig. 2a, c–e). In contrast, in the occipital cortex, a control brain region unaffected in ALS, we did not observe any change in expression of CHMP4B, CHMP2B, or VPS4 in *C9orf72* ALS/FTD or sALS neuronal nuclei (Fig. 2b–e).

### ASO mediated knockdown of CHMP7 mitigates VPS4 pathology in C9orf72 and sALS neuronal nuclei

**Fig. 3:**
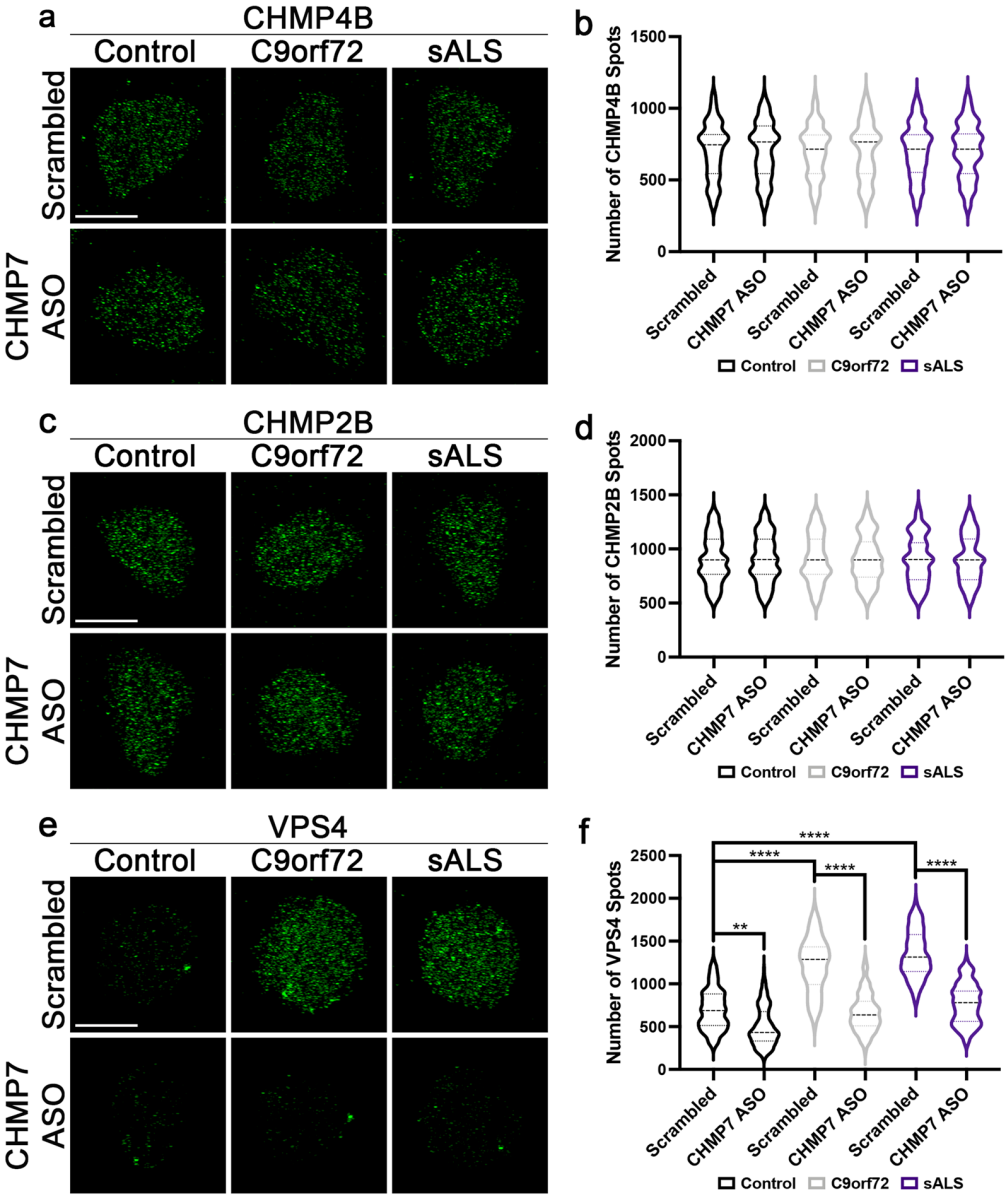
Nuclear expression of VPS4 is dependent upon CHMP7 in C9orf72 and sALS iPSNs. **a**, **c**, **e** Maximum intensity projections from SIM imaging of CHMP4B (**a**), CHMP2B (**c**), and VPS4 (**e**) in nuclei isolated from control, *C9orf72*, and sALS iPSNs following 2 week exposure to 5 μM scrambled control or CHMP7 ASO. Treatment as indicated on left, genotype and antibody as indicated on top. **b**, **d**, **f** Quantification of CHMP4B (**b**), CHMP2B (**d**), and VPS4 (**f**) spots. n = 5 control, 5 *C9orf72*, and 5 sALS iPSC lines, 50 NeuN + nuclei per line/treatment. Two-way ANOVA with Tukey’s multiple comparison test was used to calculate statistical significance. **p < 0.01, ****p < 0.0001. Scale bar = 5 μm

The nuclear relocalization of CHMP7 from the cytoplasm to the nucleus has been proposed to initiate the recruitment of additional ESCRT-III pathway components culminating in scission and removal of NPC and NE components by VPS4 [6, 23, 25, 29, 30, 32, 34]. We have previously shown that antisense oligonucleotide (ASO) mediated knockdown of CHMP7 not only alleviates the aberrant nuclear accumulation of CHMP7, but also robustly mitigates NPC injury and downstream NPC and TDP-43 dysfunction in *C9orf72* and sALS iPSNs [4]. To test whether increased nuclear CHMP7 impacted the nuclear distribution and expression of downstream components of the ESCRT-III pathway in human neurons, we treated control and ALS iPSNs with CHMP7 targeting ASOs [4] for 2 weeks after the emergence of CHMP7 [4] and VPS4 (Fig. 1a, d) pathology (see Materials and Methods). Using SIM, we find that ASO mediated CHMP7 knockdown has no impact on nuclear CHMP4B and CHMP2B immunoreactivity (Fig. 3a–d). In contrast, reduction of CHMP7 levels [4] significantly decreases nuclear VPS4 immunoreactivity (Fig. 3e, f) suggesting that increased nuclear VPS4 expression is dependent on CHMP7.

### Impaired VPS4 function is not sufficient to alleviate NPC alterations in C9orf72 and sALS neuronal nuclei

**Fig. 4:**
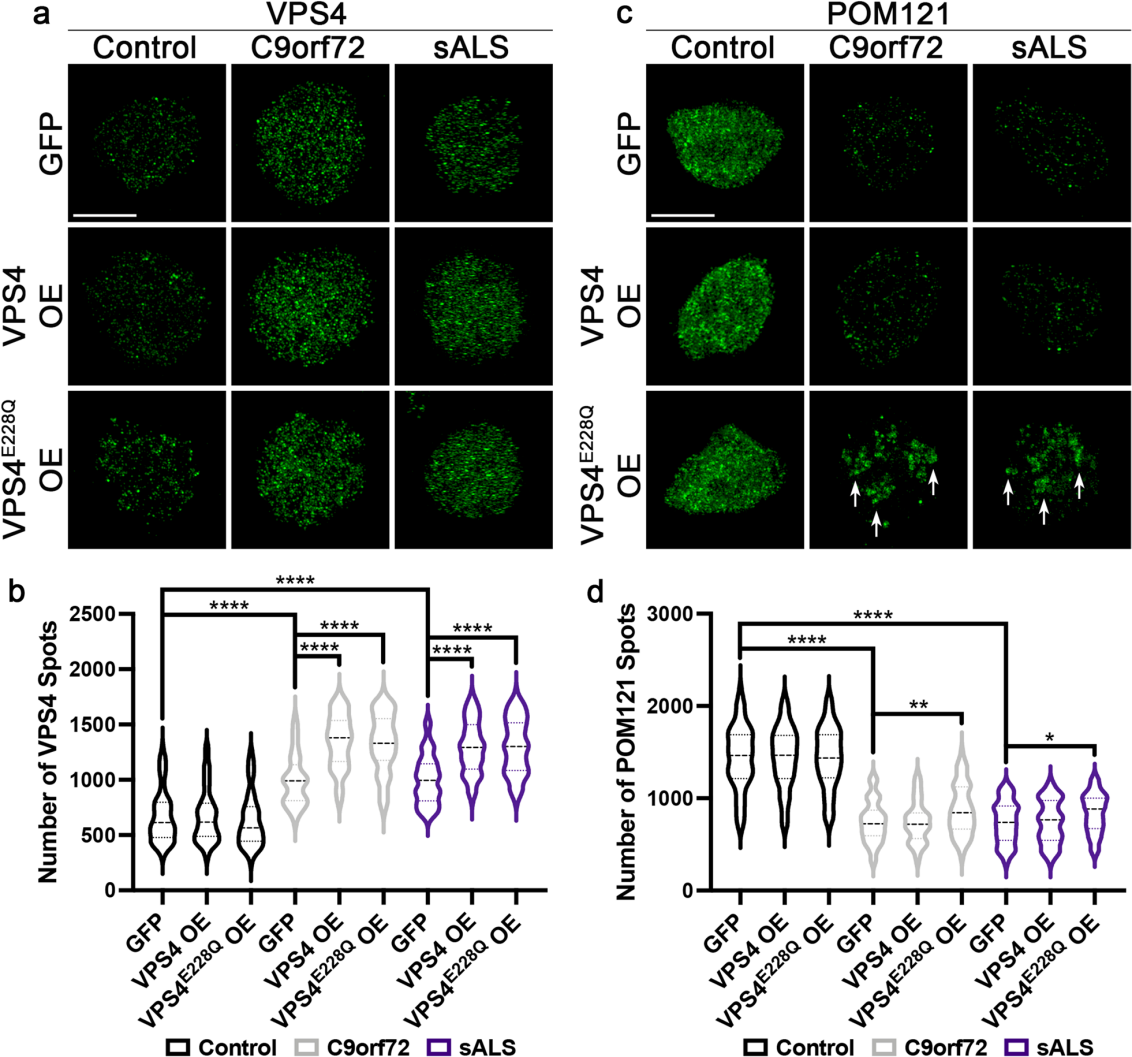
Overexpression of a dominant negative VPS4 increases nuclear POM121 spots but does not restore their distribution in C9orf72 and sALS iPSNs. **a**, **c** Maximum intensity projections from SIM imaging of VPS4 (**a**) and POM121 (**c**) in nuclei isolated from control, *C9orf72*, and sALS iPSNs overexpressing GFP or GFP tagged VPS4 variants. Overexpression as indicated on left, genotype and antibody as indicated on top. Arrows (**c**) indicate uneven distribution of POM121 observed following overexpression of dominant negative VPS4 (VPS4^E228Q^) in ALS nuclei. **b**, **d** Quantification of VPS4 (**b**) and POM121 (**d**) spots. n = 4 control, 4 *C9orf72*, and 4 sALS iPSC lines, 50 GFP + nuclei per line/overexpression. Two-way ANOVA with Tukey’s multiple comparison test was used to calculate statistical significance. *p < 0.05, **p < 0.01, ****p < 0.0001. Scale bar = 5 μm

We have previously reported that substantial reduction of the Nup POM121 from NPC and nucleoplasm is an early and consistent injury to the NPC [5] as a result of aberrant nuclear accumulation of CHMP7 [4]. As VPS4 is a AAA-ATPase that facilitates the removal of NPC and NE components from the nucleus and nuclear membrane [23, 29, 32–34], we hypothesized that increased nuclear VPS4 expression might also functionally contribute to NPC injury in *C9orf72* ALS/FTD and sALS neurons. To test this, we overexpressed GFP tagged wildtype VPS4 or a GFP tagged dominant negative variant of VPS4 (VPS4^E228Q^) that has previously been shown to impair ESCRT dependent release events [31] and performed SIM and immunostaining for POM121 in nuclei isolated from control and ALS iPSNs. Overexpression of VPS4 variants increased the nuclear expression of VPS4 as detected by immunostaining in *C9orf72* and sALS, but not control iPSNs (Fig. 4a, b) suggesting that nuclear recruitment of VPS4 is not “hyper activated” in the context of a wildtype human neuron. Consistent with a function for VPS4 downstream of CHMP7, VPS4^E228Q^ overexpression only partially restored the nuclear expression of POM121 in *C9orf72* and sALS iPSNs (Fig. 4c, d). Intriguingly, overexpression of wildtype VPS4 had no impact on nuclear POM121 immunoreactivity in *C9orf72* and sALS iPSNs (Fig. 4c, d) suggesting that simply increasing nuclear VPS4 levels is not sufficient to enhance NPC injury. Compared to control nuclei, the distribution of POM121 appears to be abnormal in *C9orf72* ALS/FTD and sALS nuclei overexpressing VPS4^E228Q^ (Fig. 4c) for reasons that remain unclear. Notably, Trim21 mediated knockdown (Trim Away, [2]) of endogenous VPS4 protein was toxic to iPSNs perhaps as a result of its functions beyond nuclear envelope and NPC homeostasis [17, 29]. Collectively, these data suggest that in contrast to CHMP7 knockdown [4], impaired VPS4 function is not sufficient to restore the expression and distribution of specific Nups within *C9orf72* ALS/FTD and sALS nuclei.

## Discussion

Nuclear pore complex injury, pathological cytoplasmic accumulations of specific Nups, and defects and nucleocytoplasmic transport have now been reported as prominent pathological features of multiple neurodegenerative diseases including ALS/FTD, AD, and HD [4, 5, 7, 10, 35]. However, the molecular mechanisms leading to these disruptions are still poorly understood. We have recently established that aberrant nuclear accumulation of the ESCRT-III protein CHMP7 is sufficient to initiate a reduction in specific Nups, beginning with POM121, from the NPC and nucleoplasm of *C9orf72* ALS/FTD and sALS human neuronal nuclei [4]. Ultimately, this nuclear pore injury impacts NCT and subsequent TDP-43 function and localization and downstream neuronal survival in response to glutamate stress [5]. To further characterize ESCRT-III protein pathology and the contribution to NPC injury in familial and sporadic ALS, we now report that VPS4, but not CHMP4B or CHMP2B, is increased in a CHMP7 dependent manner in *C9orf72* ALS/FTD and sALS human neuronal nuclei. Together, our data support a critical role for and highlight the complexity of the ESCRT-III pathway in NPC injury in ALS/FTD.

A role for the ESCRT-III pathway in NPC and NE surveillance and homeostasis and NE sealing has recently been characterized in yeast and non-neuronal mammalian cells. Specifically, loss of nuclear/cytoplasmic compartmentalization and exposure of the inner nuclear membrane results leads to slow diffusion of CHMP7 to sites of nuclear injury. Nuclear recruitment of CHMP7 “activates” ESCRT-III mediated degradation of damaged nuclear components thereby initiating nuclear repair. Notably, the AAA-ATPase VPS4 facilitates the removal of damaged nuclear envelope or NPC components via scission [15, 17, 23, 26, 29, 32, 34]. Following removal from the nuclear envelope, these proteins and/or protein complexes can be degraded via the proteasome or lysosome (autophagy) [12, 24, 32]. In contrast to these studies, we have previously shown that nuclear accumulation of CHMP7 is not responding to NPC injury, but instead initiates NPC injury in *C9orf72* ALS/FTD and sALS human neurons. Further, reducing CHMP7 levels via ASO alleviates NPC injury and downstream defects in NCT, TDP-43 function, and neuronal survival [4]. In our current study, we show that although VPS4 is recruited to neuronal nuclei in a CHMP7 dependent manner, impaired VPS4 function is not sufficient to mitigate POM121 alterations in *C9orf72* ALS/FTD and sALS neurons (Fig. 4). While future work is still needed to understand the nature of the nuclear POM121 "accumulations" that result from overexpression of a dominant negative variant of VPS4 (Fig. 4), it is plausible that these structures represent degradation intermediates.

It has been proposed that the ESCRT-III proteins CHMP4B and CHMP2B may contribute to the piecemeal turnover of specific Nups during NPC aging [25]. Although we do not observe nuclear CHMP4B and CHMP2B pathology in *C9orf72* ALS/FTD or sALS human neurons (Figs. 1, 2), we cannot rule out the possibility that the cytoplasmic functions of these ESCRT-III proteins are impaired in ALS pathogenesis. As the ESCRT-III pathway has been implicated in multiple cytoplasmic cellular processes including exosomal and endosomal trafficking and neuronal pruning [17, 29], future studies are necessary to evaluate the extent of ESCRT-III dysfunction in ALS and related neurodegenerative diseases. Further, given that a nuclear signal can be detected for CHMP4B and CHMP2B (Figs. 1, 2, Additional file 1: S1) it remains possible that although overall levels are unchanged, these proteins may functionally contribute to Nup degradation and homeostasis in aging neurons. Interestingly, CHMP2B mutations have been identified as causative of FTD [21]. These mutations are thought to impair the cytoplasmic endosomal and lysosomal functions of CHMP2B [1, 11, 14, 19, 27, 28]. Future studies are necessary to evaluate NPC homeostasis in the context of CHMP4B and/or CHMP2B knockdown in human neurons and in the context of CHMP2B mutations.

## Conclusions

Collectively, our data suggest that like CHMP7 [4], the nuclear expression of the ESCRT-III protein VPS4 is pathologically increased in *C9orf72* and sALS human neurons. However, the nuclear localization, distribution, and expression of the ESCRT-III pathway proteins CHMP4B and CHMP2B is unaffected in *C9orf72* ALS/FTD and sALS human neurons. Moreover, impairment of VPS4 function does not alleviate NPC injury and knockdown of VPS4 is toxic in *C9orf72* and sALS iPSNs. While the disease associated increase in nuclear levels of VPS4 is dependent upon nuclear accumulation of CHMP7, this data is in stark contrast to our previous report defining the therapeutic potential for CHMP7 knockdown in the mitigation of multiple neuronal pathophysiological defects [4]. Thus, together, these data highlight the necessity for therapeutic targeting of the most upstream events in this early and prominent neurodegenerative cascade.

## Supplementary Information

**Additional file 1**: **Figure 1** Related to Figure 1: The expression of VPS4 is increased in C9orf72 and sALS iPSN nuclei but not whole iPSN lysates. (a-d) Western blot (a) and quantification (b-d) for CHMP4B (a-b), CHMP2B (a, c), and VPS4 (a, d) expression in control, C9orf72, and sALS iPSN lysates. Antibodies as indicated on right, genotype as indicated on bottom. GAPDH was used as a loading control. n = 4 control, 4 C9orf72, and 4 sALS iPSC lines. One-way ANOVA with Tukey’s multiple comparison test was used to calculate statistical significance. (e-h) Western blot (e) and quantification (f-h) for CHMP4B (e-f), CHMP2B (e, g), and VPS4 (e, h) expression in nuclei isolated from control, C9orf72, and sALS iPSNs. Antibodies as indicated on right, genotype as indicated on bottom. Lamin B1 was used as a loading control. n = 4 control, 4 C9orf72, and 4 sALS iPSC lines. One-way ANOVA with Tukey’s multiple comparison test was used to calculate statistical significance. ** p < 0.01.

**Additional file 2.**

## Data Availability

All iPSC lines are available from the Cedars Sinai Answer ALS cell line bank (https://www.cedars-sinai.edu/Research/Research-Cores/Induced-Pluripotent-Stem-Cell-Core-/Answer-ALS-Project.aspx) or through the Answer ALS Data portal (https://dataportal.answerals.org/home).
