# Supplementary figures and images for: The ESCRT-III protein VPS4, but not CHMP4B or CHMP2B, is pathologically increased in familial and sporadic ALS neuronal nuclei
Source: Acta Neuropathol Commun. 2021 Jul 19;9:127. doi: 10.1186/s40478-021-01228-0 (PMC8287756; doi:10.1186/s40478-021-01228-0)

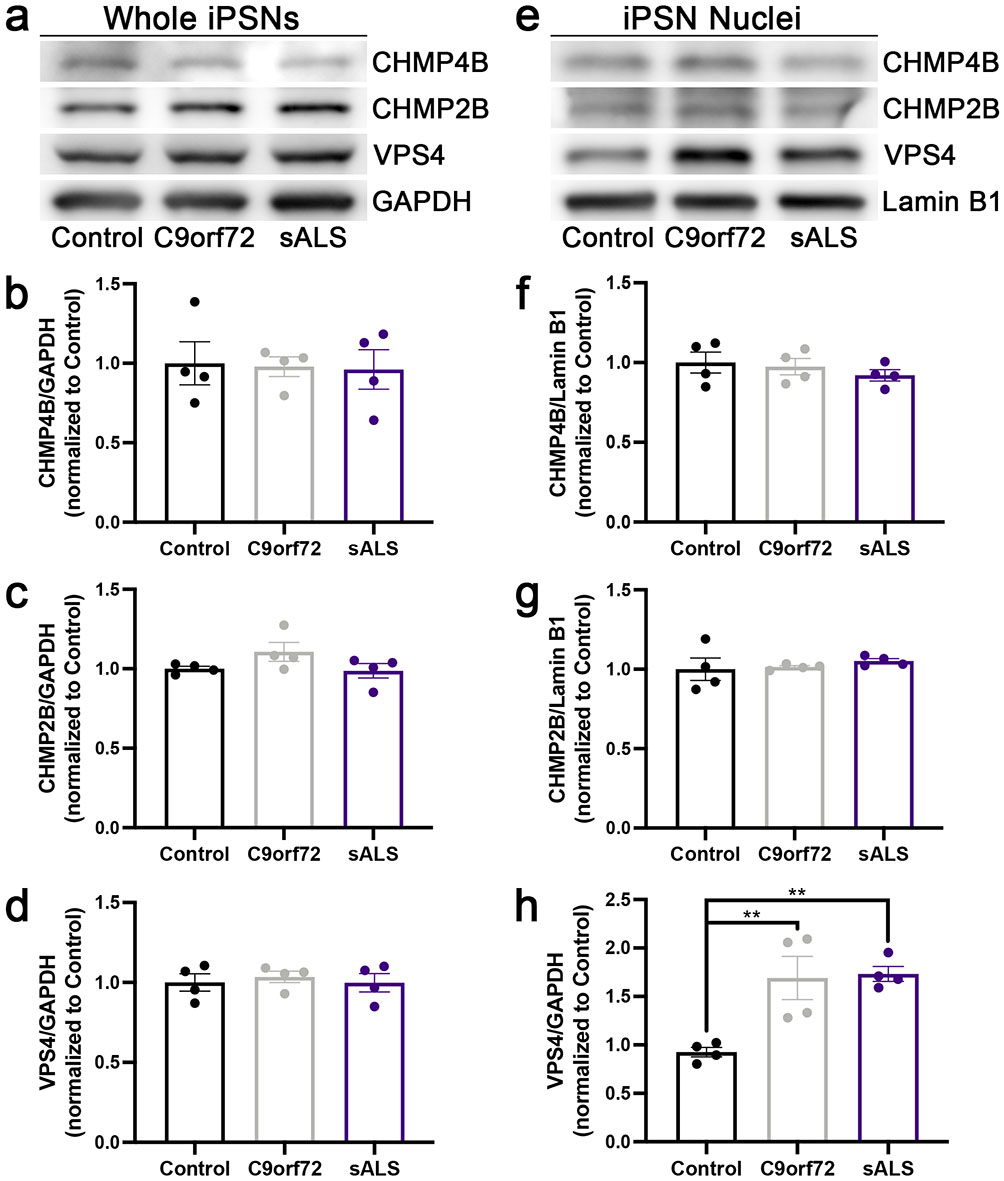

Supplement: Supplementary file 1 — Additional file 1: Figure 1 Related to Figure 1: The expression of VPS4 is increased in C9orf72 and sALS iPSN nuclei but not whole iPSN lysates. (a-d) Western blot (a) and quantification (b-d) for CHMP4B (a-b), CHMP2B (a, c), and VPS4 (a, d) expression in control, C9orf72, and sALS iPSN lysates. Antibodies as indicated on right, genotype as indicated on bottom. GAPDH was used as a loading control. n = 4 control, 4 C9orf72, and 4 sALS iPSC lines. One-way ANOVA with Tukey’s multiple comparison test was used to calculate statistical significance. (e-h) Western blot (e) and quantification (f-h) for CHMP4B (e-f), CHMP2B (e, g), and VPS4 (e, h) expression in nuclei isolated from control, C9orf72, and sALS iPSNs. Antibodies as indicated on right, genotype as indicated on bottom. Lamin B1 was used as a loading control. n = 4 control, 4 C9orf72, and 4 sALS iPSC lines. One-way ANOVA with Tukey’s multiple comparison test was used to calculate statistical significance. ** p < 0.01. [file 40478_2021_1228_MOESM1_ESM.jpg]
