# Supplementary material for: The ESCRT-III protein VPS4, but not CHMP4B or CHMP2B, is pathologically increased in familial and sporadic ALS neuronal nuclei
Source: Acta Neuropathol Commun. 2021 Jul 19;9:127. doi: 10.1186/s40478-021-01228-0 (PMC8287756; doi:10.1186/s40478-021-01228-0)
Supplement: Supplementary file 2 — Additional file 2. [file 40478_2021_1228_MOESM2_ESM.docx]

**Supplemental Table 1: Demographic Information for iPSC Lines**

| **iPSC Line Name** | **Source** | **Clinical Diagnosis** | **Age at Time of Collection** | **Sex** | **Origin** |
| --- | --- | --- | --- | --- | --- |
| EDi036-A | Cedars-Sinai | Non-neurologic control | 79 | Female | PBMC |
| EDi037-A | Cedars-Sinai | Non-neurologic control | 79 | Male | PBMC |
| EDi029-A | Cedars-Sinai | Non-neurologic control | 80 | Male | PBMC |
| EDi034-A | Cedars-Sinai | Non-neurologic control | 79 | Female | PBMC |
| CS1ATZ | Cedars-Sinai | Non-neurologic control | 60 | Male | PBMC |
| CS8PAA | Cedars-Sinai | Non-neurologic control | 58 | Female | PBMC |
| EDi043-A | Cedars-Sinai | Non-neurologic control | 80 | Male | PBMC |
| CS0002 | Cedars-Sinai | Non-neurologic control | 51 | Male | PBMC |
| CS9XH7 | Cedars-Sinai | Non-neurologic control | 53 | Male | PBMC |
| CS0BUU | Cedars-Sinai | C9orf72 ALS | 63 | Female | PBMC |
| CS7VCZ | Cedars-Sinai | C9orf72 ALS | 64 | Male | PBMC |
| CS0LPK | Cedars-Sinai | C9orf72 ALS | 67 | Male | PBMC |
| CS6ZLD | Cedars-Sinai | C9orf72 ALS |  | Female | PBMC |
| CS8KT3 | Cedars-Sinai | C9orf72 ALS | 60 | Male | PBMC |
| CS2YNL | Cedars-Sinai | C9orf72 ALS | 60 | Male | PBMC |
| CS0NKC | Cedars-Sinai | C9orf72 ALS | 52 | Female | PBMC |
| CS6CLW | Cedars-Sinai | C9orf72 ALS |  | Male | PBMC |
| CS6UC9 | Cedars-Sinai | C9orf72 ALS | 54 | Male | PBMC |
| 59-1 | K. Talbot | Isogenic Correction of OXC9-02 | 62 | Female | Fibroblast |
| OXC9-02-02 | K. Talbot | C9orf72 | 62 | Female | Fibroblast |
| CS3XLK | Cedars-Sinai | sALS | 55 | Female | PBMC |
| CS5JPF | Cedars-Sinai | sALS | 55 | Female | PBMC |
| CS8JGP | Cedars-Sinai | sALS | 61 | Male | PBMC |
| CS6MBU | Cedars-Sinai | sALS |  | Male | PBMC |
| CS1KL3 | Cedars-Sinai | sALS | 71 | Female | PBMC |
| CS9GXD | Cedars-Sinai | sALS | 68 | Male | PBMC |
| CS0JGZ | Cedars-Sinai | sALS | 56 | Male | PBMC |
| CS2EVP | Cedars-Sinai | sALS | 69 | Male | PBMC |
| CS6PYD | Cedars-Sinai | sALS | 69 | Female | PBMC |
| CS5ZHY | Cedars-Sinai | sALS | 64 | Male | PBMC |

**Supplemental Table 2: Demographic Information for Postmortem Human Tissue**

|  | **Clinical Diagnosis** | **Age of Death** | **Sex** |
| --- | --- | --- | --- |
| **Control** | Non-neurologic control | 70 | Female |
| **Control** | Non-neurologic control | 92 | Female |
| **Control** | Non-neurologic control | 72 | Male |
| **Control** | Non-neurologic control | 37 | Female |
| **Control** | Non-neurologic control | 50 | Male |
| **Control** | Non-neurologic control | 52 | Male |
| **C9orf72** | C9orf72 ALS/FTD | 59 | Male |
| **C9orf72** | C9orf72 ALS | 72 | Male |
| **C9orf72** | C9orf72 ALS | 69 | Female |
| **C9orf72** | C9orf72 ALS/FTD | 61 | Female |
| **C9orf72** | C9orf72 ALS | 68 | Female |
| **C9orf72** | C9orf72 ALS/FTD | 74 | Male |
| **sALS-1** | sALS | 69 | Male |
| **sALS-2** | sALS | 68 | Female |
| **sALS-3** | sALS | 69 | Male |
| **sALS-4** | sALS | 67 | Female |
| **sALS-5** | sALS | 59 | Female |
| **sALS-6** | sALS | 68 | Male |
| **sALS-7** | sALS | 71 | Female |
| **sALS-8** | sALS | 66 | Male |
| **sALS-9** | sALS | 70 | Female |

**Supplemental Table 3: Antibody Information**

| **Primary Antibodies** | | | | |
| --- | --- | --- | --- | --- |
| **Antibody** | **Source** | | **Catalog Number** | **Application and Concentration** |
| Rabbit Anti-CHMP4B | Proteintech | | 13683-1-AP | IF: 1/250  Western: 1/1000 |
| Rabbit Anti-CHMP2B | Thermo Fisher Scientific | | PA531128 | IF: 1/250  Western: 1/1000 |
| Mouse Anti-VPS4 | Santa Cruz Biotechnology | | sc-133122 | IF: 1/50  Western: 1/250 |
| Rabbit Anti-POM121 | Novus Biologicals | | NBP2-19890 | IF: 1/250 |
| Chicken Anti-NeuN | Millipore | | ABN91 | IF: 1/500 |
| Chicken Anti-GFP | Millipore | | AB16901 | IF: 1/1000 |
| Rabbit Anti-Lamin B1 | Abcam | | ab16048 | Western: 1/1000 |
| Mouse Anti-GAPDH | Life Technologies | | AM4300 | Western: 1/10,000 |
| **Secondary Antibodies** | | | | |
| Goat Anti-Mouse Alexa 488 | Invitrogen | A11029 | | IF: 1/1000 |
| Goat Anti-Rabbit Alexa 488 | Invitrogen | A11034 | | IF: 1/1000 |
| Goat Anti-Chicken Alexa 488 | Invitrogen | A11039 | | IF: 1/1000 |
| Goat Anti-Mouse Alexa 568 | Invitrogen | A11031 | | IF: 1/1000 |
| Goat Anti-Rabbit Alexa 568 | Invitrogen | A11036 | | IF: 1/1000 |
| Goat Anti-Chicken Alexa 647 | Invitrogen | A21449 | | IF: 1/1000 |
| Donkey Anti-Rabbit IgG HRP | Thermo Fisher Scientific | 45-000-682 | | Western: 1/5000 |
| Horse Anti-Mouse IgG HRP | Cell Signaling | 7076S | | Western: 1/5000 |
